# Supplementary material for: Clinical Implication of Liquid Biopsy in Colorectal Cancer Patients Treated with Metastasectomy
Source: Cancers (Basel). 2021 May 6;13(9):2231. doi: 10.3390/cancers13092231 (PMC8125778; doi:10.3390/cancers13092231)
Supplement: Supplementary file 1 [file cancers-13-02231-s001.zip › Supplement Table 1.pdf]

Supplementary Table S1.

Ion AmpliSeq™ Comprehensive Cancer Panel target gene list (50 genes)

ABL1	*BRAF*	*EGFR*	*FGFR1*	*GNAQ*	*IDH2*	*KRAS*	*NPM1*	*PTPN11*	*SMO*
AKT1	*CDH1*	*ERBB2*	*FGFR2*	*GNAS*	*JAK2*	*MET*	*NRAS*	*RB1*	*SRC*
ALK	*CDKN2A*	*ERBB4*	*FGFR3*	*HNF1A*	*JAK3*	*MLH1*	*PDGFRA*	*RET*	*STK11*
APC	*CSF1R*	*EZH2*	*FLT3*	*HRAS*	*KDR*	*MPL*	*PIK3CA*	*SMAD4*	*TP53*
ATM	*CTNNB1*	*FBXW7*	*GNA11*	*IDH1*	*KIT*	*NOTCH1*	*PTEN*	*SMARCB1*	*VHL*

Strategically designed to interrogate coding DNA sequences and splice variants across multiple gene families simultaneously, this pathway-based gene selection profiles the mutational spectrum in cancer driver genes and drug targets along with signaling cascades, apoptosis genes, DNA repair genes, transcription regulators, inflammatory response genes, and growth factor genes in a single assay. This panel targets all 50 genes targeted in the focused Ion AmpliSeq™ Cancer Panel v2.
